# Endovascular management of an isolated common iliac artery aneurysm: a case report

**DOI:** 10.11604/pamj.2021.40.69.30814

**Published:** 2021-09-30

**Authors:** Imtinene Ben Mrad, Melek Ben Mrad, Sobhi Mleyhi, Rim Miri, Ihsen Zairi, Yassine Khaddar, Mohamed Ben Hammamia, Raouf Denguir

**Affiliations:** 1 Cardiology Department, Habib Thameur Hospital, Tunis, Tunisia,; 2 Cardiovascular Surgery Department, Rabta Hospital, Tunis, Tunisia

**Keywords:** Iliac artery, aneurysm, endovascular, embolization, case report

## Abstract

Isolated iliac artery aneurysms are rare, and treatment by conventional surgery gives good results. Endovascular repair of such aneurysms has recently become the preferred form of treatment, provided the appropriate anatomy for endovascular repair exists. We report the case of a 60-year-old man admitted in our department for an aneurysm of the left primitive iliac artery revealed by intermittent claudication and treated by a covered stent after embolization of the hypogastric artery by an Amplatzer Vascular Plug with a good result. This case highlights the importance of preservation of the collaterals of the hypogastric artery when you treat such entity; in order to avoid transient gluteal claudication and sexual dysfunction.

## Introduction

Isolated common iliac artery (CIA) aneurysms are rare. In order to prevent the risk of rupture, treatment of these aneurysms is only considered when the diameter exceeds three centimeters. Rupture of a CIA aneurysm is associated with an immediate mortality rate of around 70% [1]. Percutaneous techniques are effective alternatives in selected cases; however, they require a suitable anatomy in order to achieve a stable and durable repair over time. Preservation of the internal iliac artery (IIA) is only possible when the distal neck is sufficiently long. In this article, we report the endovascular treatment of a CIA aneurysm using a covered stent with embolization of the hypogastric artery by an Amplatzer Vascular Plug. This case is singular for the reasons: the use a conic covered stent and the use of an Amplatzer for the embolization allowing the preservation of collaterals of the hypogastric artery and so avoiding gluteal claudication and sexual dysfunction.

## Patient and observation

**Patient information:** we report the observation of a 60-year-old Tunisian man with history of smoking, hypertension, diabetes, and aortocoronary bypass surgery who presented in our department 4 years ago with mild intermittent claudication of the left lower limb with a walking perimeter equal to 500 meters.

**Clinical findings:** clinical examination found an absence of popliteal and distal pulses on the left lower limb. Arterial pressure was 120/80mmHg. Pulmonary and cardiac examinations were normal and so was the rest of the clinical assessment.

**Figure 1:**
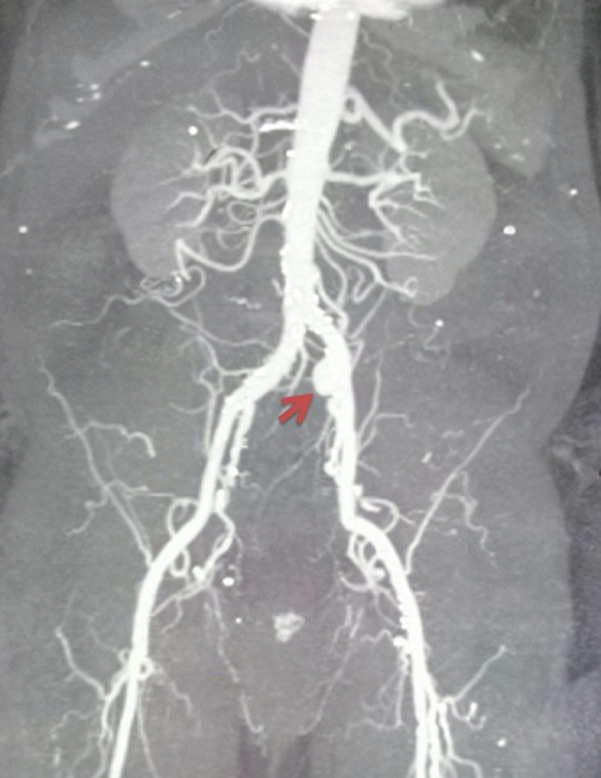
computerized tomography (CT) scan showing an isolated aneurysm of the left common iliac artery

**Diagnostic assessment:** US Duplex objectified an aneurysm of the left CIA measuring 35 mm in diameter and total thrombosis of the left superficial femoral artery. A CT scan confirmed the same diagnosis (Figure 1).

**Figure 2:**
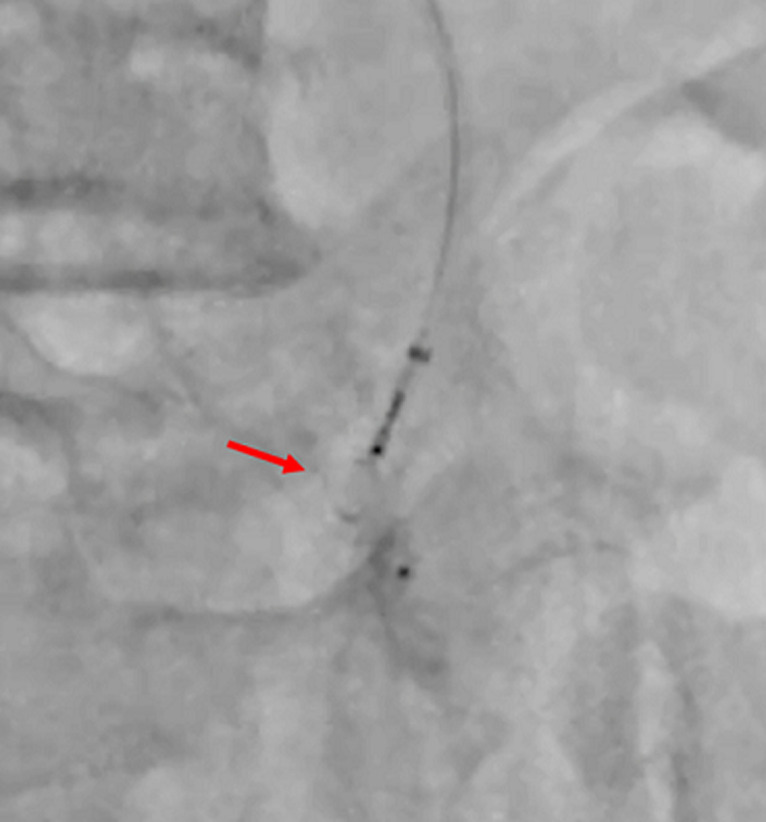
perioperative angiography showing the deployment of an Amplatzer vascular plug at the origin of the left hypogastric artery (red arrow)

**Figure 3:**
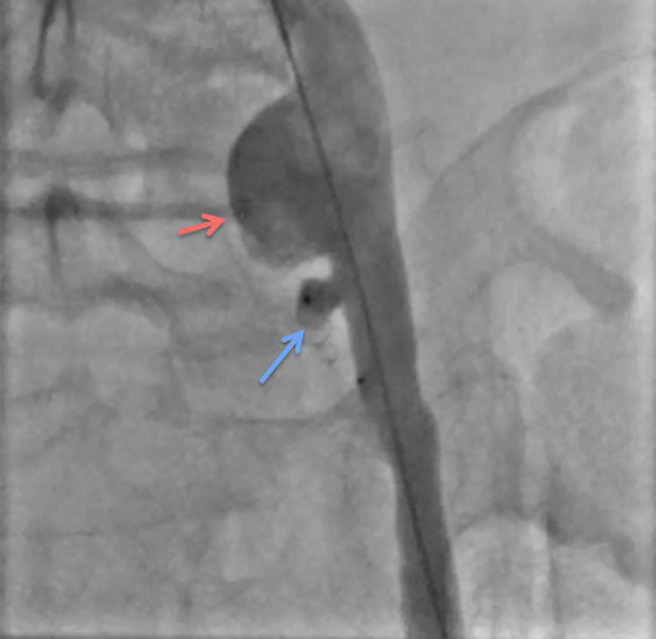
perioperative angiography showing the total occlusion of the left hypogastric artery (blue arrow) and the aneurysm of the common iliac artery (red arrow)

**Figure 4:**
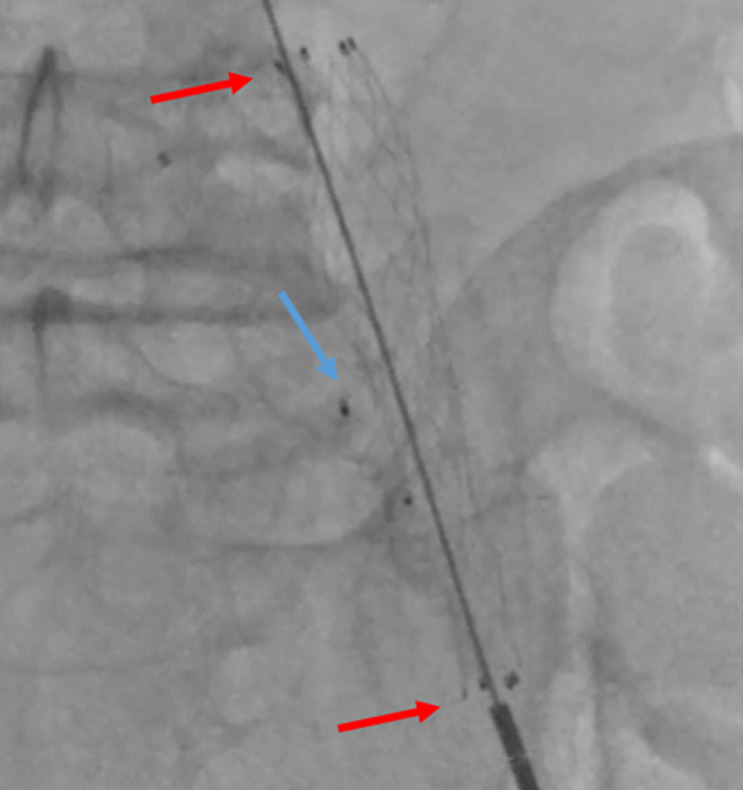
perioperative angiography showing the deployment of the fluency stent (red arrows) and the presence of the Amplatzer vascular plug outside (blue arrow)

**Figure 5:**
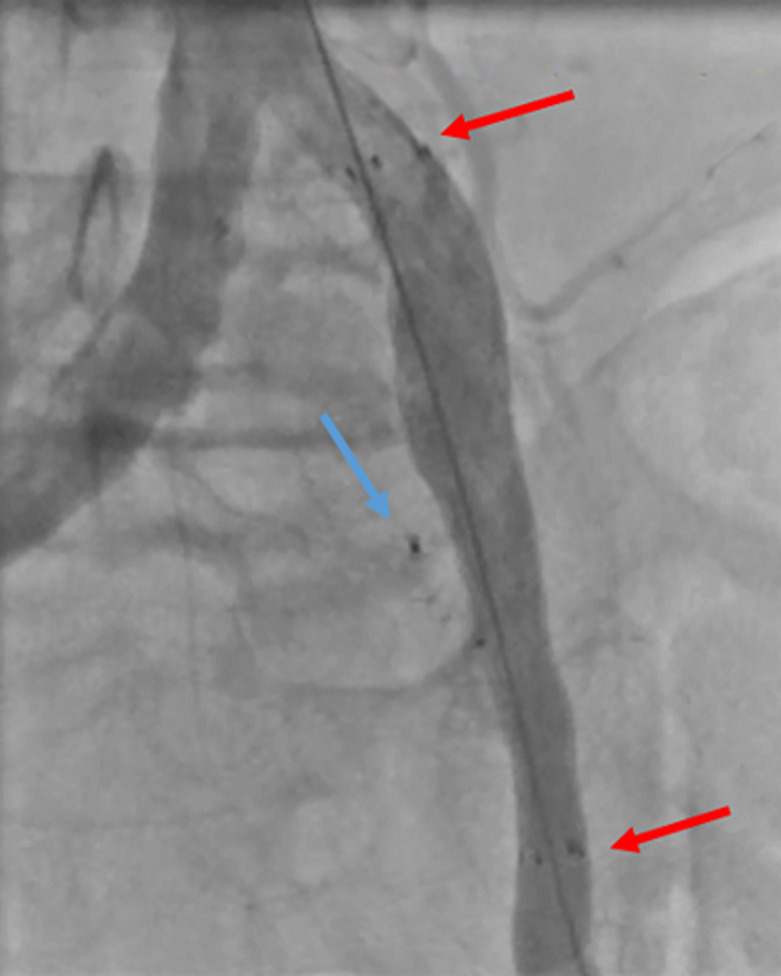
final angiography showing the total exclusion of the aneurysm (red arrows) total occlusion of the left hypogastric artery (blue arrow)

**Therapeutic intervention:** given the patient's high obesity (BMI > 36) and several comorbidities, we opted for endovascular management of the iliac aneurysm. The patient was taken to a hemodynamic suite. After a right groin retrograde access, a 45cm crossover 6F sheath was placed into the left CIA using a UF catheter. The first angiogram confirmed the diagnosis of an aneurysm of the iliac artery. After a selective catheterization of the left internal iliac artery, we deployed an 8-mm-diameter × 7-mm-long nitinol-based Amplatzer Vascular Plug (AGA Medical Corp, Golden Valley, Minn) on the origin of this artery (Figure 2). First angiographic control showed the total occlusion of the hypogastric artery (Figure 3). Secondly, a surgical access to the common femoral artery was achieved by an open dissection of the groin under local anesthesia. After a retrograde left femoral puncture, and setting up an 11F sheath, we passed the lesion with a 0.035 hydrophilic guide wire with the help of a 5F Bernstein diagnostic catheter. We exchanged the first wire by a stiff 0.035 one which was advanced through the iliac artery to the aorta. We then deployed a 13.5-mm-diameter, 80-mm-long self-expanding covered stent (Fluency; Bard Peripheral Vascular) (Figure 4). Final angiogram showed the exclusion of the aneurysm with patency of the iliac conduit (Figure 5). Adjunctive therapy after the procedure included 75 mg Clopidogrel per day for 6 months and 100 mg acetylsalicylic acid per day for life.

**Follow-up and outcomes:** the patient was discharged without complications. For his intermittent claudication, we decided to keep him under only medical treatment due to his mild level of discomfort. Follow-up computed tomography after 1 month showed stent patency and total exclusion of the aneurysm. Three year after, control distinctiveness, uniformity and stability (DUS) showed correct position of the stent-graft, total exclusion, decrease in size of the aneurysm diameter, and patency of the left iliac artery.

**Patient consent:** a written informed consent was obtained from the patient for the publication of details, which can include photographs and/or videos and/or case history to be published in any printed/online journals.

## Discussion

Common iliac artery (CIA) aneurysms are rare. The main etiology is atherosclerosis, while other reports include such diverse origins as pregnancy, infection, and iatrogenic trauma [1]. They account for 2.2% to 7% of all intra-abdominal aneurysms [1, 2], and their frequency in a Swedish autopsy series was 0.03% [3]. There is a clear predominance in males, as the sex ratio varies between 5: 1 and 25: 1 in different studies [1].

The main risks of these aneurysms are embolic complications and rupture. The operative indication is retained if the aneurysm is symptomatic and/or the diameter exceeds 30-35 mm [4]. Early diagnosis is critical, because the rate of rupture increases with aneurysmal size, especially if the diameter exceeds 5cm [5]. During evolution, rupture may occur in 33 to 38% of cases, and the mortality rate reported in the literature for ruptured iliac aneurysms is 58% [6]. Therefore, elective intervention is recommended whenever the aneurysm reaches more than 3 cm in diameter [6].

Open repair remains the standard procedure for iliac aneurysm treatment. Currently, graft interposition coupled with endoaneurysmorrhaphy is the most commonly employed technique and remains the gold standard. It is a standardized procedure, but can be difficult to perform especially in obese patients or in cases of hostile abdomen. Elective operative management was undertaken in 26 patients with a mortality rate of 11% in the large Richardson Cohort [1].

In the last two decades, endovascular treatment has become an alternative to conventional surgery, as it allows for shorter hospitalization and can be performed under local anesthesia. Endovascular treatment of CIA aneurysms has become the first-line treatment whenever anatomical conditions are suitable. It should be noted that proximal and distal necks (> 1.5 cm in length) without mural thrombus are necessary to have a correct landing-zone [7]. In addition, the diameter of the proximal sealing zone is often significantly larger than the diameter of the distal sealing zone, which is the external iliac artery (EIA) in most cases.

Different types of stent-grafts have been used to treat these iliac aneurysms. Some authors have described endovascular treatment of isolated aneurysms of the CIA using iliac extensions of aorto-iliac endoprosthesis [8]. These extensions have a funnel-shaped form with a decreasing diameter from top to bottom, which allows them to be placed at the level of the EIA, especially in situations when the origin of the hypogastric artery must be covered by the lack of an adequate distal landing zone at the level of the CIA. These extensions are made of PTFE with an external nitinol stent, are very flexible, and therefore adapt easily to different configurations of the iliac artery anatomy [8].

In our case, the distal neck in the CIA was 1 cm, so we decided to choose the EIA as a distal landing zone. We considered, in order avoiding a late possible type II endoleak by backflow from the IIA, that it is preferable to first embolize the latter with an Amplatzer Vascular Plug. Embolization was performed at the origin of the IIA to preserve flow in the different distal branches. The Wallgraft stent is flexible and can be adapted to different diameters of the vessel, thus allowing a concomitant landing at the CIA and EIA despite a 2mm difference in diameter between the two arteries in our case. However, this type of stent requires the use of an 11 F sheath, which led us to perform a surgical approach to the femoral artery because percutaneous closure systems are not available in our department.

Embolization of the internal iliac artery is unavoidable in most cases. It may be associated in 30% of cases with moderate and transient gluteal claudication and sexual dysfunction, particularly when thrombosis extends to the collateral of the hypogastric artery [9]. However, this risk remains very low, especially when only the origin is occluded. Embolization with an Amplatzer device is a good alternative to conventional coils, since it allows precise deployment to preserve the hypogastric bifurcation and therefore the contralateral pelvic flow [10]. A stent-graft with an incorporated iliac bifurcation for insertion of a covered stent-graft into the IIA is another promising solution for preserving flow to the IIA [8].

The results of endovascular treatment appear to be good. Pitoulias *et al*. [6] in a retrospective multicentric study involving 58 patients comparing endovascular to surgical treatment of isolated CIA aneurysms reported a 3-year primary permeability rate of 97% in the endovascular group. No secondary intervention was required in either group throughout the follow-up period [6].

## Conclusion

Percutaneous treatment by embolization of the hypogastric artery and deployment of a covered stent graft appears to be a feasible and effective alternative to conventional surgery for the treatment of isolated CIA aneurysm whenever the anatomical conditions are suitable. The Use of an Amplatzer Vascular Plug allows the preservation of the hypogastric collaterals and so avoids the risk pf sexual dysfunction and gluteal claudication.
